# Early-Onset Convulsive Seizures Induced by Brain Hypoxia-Ischemia in Aging Mice: Effects of Anticonvulsive Treatments

**DOI:** 10.1371/journal.pone.0144113

**Published:** 2015-12-02

**Authors:** Justin Wang, Chiping Wu, Jessie Peng, Nisarg Patel, Yayi Huang, Xiaoxing Gao, Salman Aljarallah, James H. Eubanks, Robert McDonald, Liang Zhang

**Affiliations:** 1 Toronto Western Research Institute, University Health Network, Toronto, Ontario, Canada; 2 Departments of Medicine (Neurology), University of Toronto, Toronto, Ontario, Canada; 3 Department of Surgery (Neurosurgery), University of Toronto, Toronto, Ontario, Canada; 4 Department of Neuroscience, University of Lethbridge, Lethbridge, Alberta, Canada; 5 Neurology Unit, Department of Medicine, King Saud University, Riyadh, Saudi Arabia; University of Modena and Reggio Emilia, ITALY

## Abstract

Aging is associated with an increased risk of seizures/epilepsy. Stroke (ischemic or hemorrhagic) and cardiac arrest related brain injury are two major causative factors for seizure development in this patient population. With either etiology, seizures are a poor prognostic factor. In spite of this, the underlying pathophysiology of seizure development is not well understood. In addition, a standardized treatment regimen with anticonvulsants and outcome assessments following treatment has yet to be established for these post-ischemic seizures. Previous studies have modeled post-ischemic seizures in adult rodents, but similar studies in aging/aged animals, a group that mirrors a higher risk elderly population, remain sparse. Our study therefore aimed to investigate early-onset seizures in aging animals using a hypoxia-ischemia (HI) model. Male C57 black mice 18-20-month-old underwent a unilateral occlusion of the common carotid artery followed by a systemic hypoxic episode (8% O_2_ for 30 min). Early-onset seizures were detected using combined behavioral and electroencephalographic (EEG) monitoring. Brain injury was assessed histologically at different times post HI. Convulsive seizures were observed in 65% of aging mice post-HI but not in control aging mice following either sham surgery or hypoxia alone. These seizures typically occurred within hours of HI and behaviorally consisted of jumping, fast running, barrel-rolling, and/or falling (loss of the righting reflex) with limb spasms. No evident discharges during any convulsive seizures were seen on cortical-hippocampal EEG recordings. Seizure development was closely associated with acute mortality and severe brain injury on brain histological analysis. Intra-peritoneal injections of lorazepam and fosphenytoin suppressed seizures and improved survival but only when applied prior to seizure onset and not after. These findings together suggest that seizures are a major contributing factor to acute mortality in aging mice following severe brain ischemia and that early anticonvulsive treatment may prevent seizure genesis and improve overall outcomes.

## Introduction

Old age is an independent risk factor for the development of seizures and epilepsy [1, 2]. In this cohort, both hemorrhagic and ischemic stroke further exacerbates this risk [3–11]. Seizures have also been noted following cardiac arrest in association with the resultant global brain ischemia [12–15]. Regardless of the causative event, seizures are a poor prognostic factor [16–19]. However, in spite of this fact, standardized treatments and outcome assessments for post-ischemic seizures remain to be established [8–10; 15, 18, 20–21].

Seizures arising within two weeks of the initial stroke or cardiac event are generally categorized as “early-onset”. Early-onset seizures are primarily observed within 24 hours of the initial insult and are considered a medical emergency as life-threatening status epilepticus may develop [12–13, 22–23]. Early onset seizures can manifest as generalized convulsive seizures (CS) or focal/generalized non-convulsive seizures (NCS), which may require electroencephalography (EEG) for diagnosis [12–13, 23–27]. Although stroke severity and cortical involvement are recognized risk factors for seizure development following ischemic stroke [3–10, 27], the pathogenesis of post-ischemic seizures is still not well understood.

Previous experimental studies have examined early-onset CS and NCS in rodent models following either middle cerebral artery occlusion (MCAO) [28–37], hypoxia-ischemia (HI) [38–39], photothrombotic ischemia [40–41], or cardiopulmonary bypass resuscitation [42–44]. Although a few studies have investigated late-onset seizures in aged rats following either MCAO or photothrombotic ischemia [40, 45–46], there remain a paucity of information on early-onset, post-ischemic seizures in aging/aged animals.

Our recent work in aging mice following MCAO noted vigorous CS within hours post-ischemia [47]. Development of these early-onset CS was closely associated with severe brain injury and acute mortality. Anticonvulsive treatment failed to improve animal survival when given after CS occurrence. These previous observations suggest that early-onset CS are an early symptom and a poor prognostic factor in aging animals with severe brain ischemia. However, a potential confounder in our MCAO model is the possibility of iatrogenic subarachnoid hemorrhage from intraluminal suture insertion [48–50], which may induce early-onset CS independent of brain ischemia. In addition, the earliest “spontaneous” CS in our MCAO model had a mean latency of 30 min post-surgery, well before expected development of structural brain injury [51]. This raises the possibility that something other than just structural changes promotes CS generation, including but not limited to compromised cortical descending inhibition in the early phase of brain ischemia.

The HI model consists of a unilateral occlusion of the common carotid artery and subsequent systemic hypoxia [52–54]. This model has been found to successfully induce ipsilateral brain ischemia [54–56] and early-onset seizures [38–39] in adult animals. The short duration of anesthesia and relatively minor surgical procedure involved in the HI model are ideal for aging/aged animals that are generally more susceptible than younger animals to anesthetic and surgical side effects [57–58]. The lack of an intraluminal suture insertion step in the HI model also eliminates the risk of associated complications and confounders including subarachnoid hemorrhage [48–50] sometimes seen in the MCAO model. The hypoxic episode in the HI model can also be applied to free-moving animals, thereby enabling continuous behavioral state recording while minimizing the use of anesthetics and their potentially confounding effects on seizure generation. Taking these facts into consideration, we found the HI model to be suitable for examining early-onset post-ischemic seizures in aging mice and for evaluating the role of early anticonvulsive treatment.

## Materials and Methods

### Animals

Male C57BL/6 mice (Charles River, Senneville St-Constant, Quebec, Canada) were used. Twenty-four-month-old (aged) C57 mice correspond to an approximate human age of ≥70 years [59]. However, mice of this age frequently develop health-related complications including skin lesions, ear infections, and tumors [59]. We therefore chose to conduct our experiments in 18-20-month-old mice in order to successfully model brain ischemia in aging animals while minimizing the health-related complications commonly seen in older mice.

Our aging mice were housed in a vivarium that was maintained between 22–23°C with a 12-hour light on/off cycle. Food and water were accessible ad libitum. All experiments detailed here were reviewed and approved by the animal care committee of the University Health Network in accordance with the Canadian Guidelines for Animal Care. As per the guidelines, all animals with severe CS were treated with clinically appropriate anticonvulsants, and mandatory euthanization (with pentobarbital anesthesia) was performed if animals exhibited severe, refractory CS and/or presented in poor physical condition, defined as persistent immobility, lack of eating or drinking, irresponsiveness to touch, loss of the righting reflex, and/or a substantial reduction in body weight (≥20% of baseline level).

### Brain hypoxia-ischemia (HI)

The HI model was conducted as previously described [38]. Aging mice were anesthetized with 2% isoflurane, and the right common carotid artery was permanently ligated. A systemic hypoxic episode (8% O_2_ for up to 30 min) was conducted 90 min after the carotid artery occlusion after animals had fully recovered from the surgical anesthetic. The hypoxic episode was conducted in an air-tight chamber with input/output air paths and a gasket-sealed hole for the passage of EEG wires or a laser Doppler probe [60]. The inner temperature of the chamber was maintained between 32–33°C to minimize the effects of hypoxic hypothermia [38, 54, 60–62].

We aimed to expose each animal to a 30-minute hypoxic episode. However, the hypoxia was terminated immediately in line with our animal care guidelines for any mice with observable signs of impending respiratory arrest or vigorous convulsions. Despite taking this precaution, 6 aging mice still died during hypoxia. These animals were excluded from data analysis. Control mice received either a sham surgery (isolation of the common carotid artery without subsequent occlusion) or exposure to a 30-min hypoxic episode without occlusion of the common carotid artery.

### Electroencephalographic (EEG) recordings

**Fig 1:**
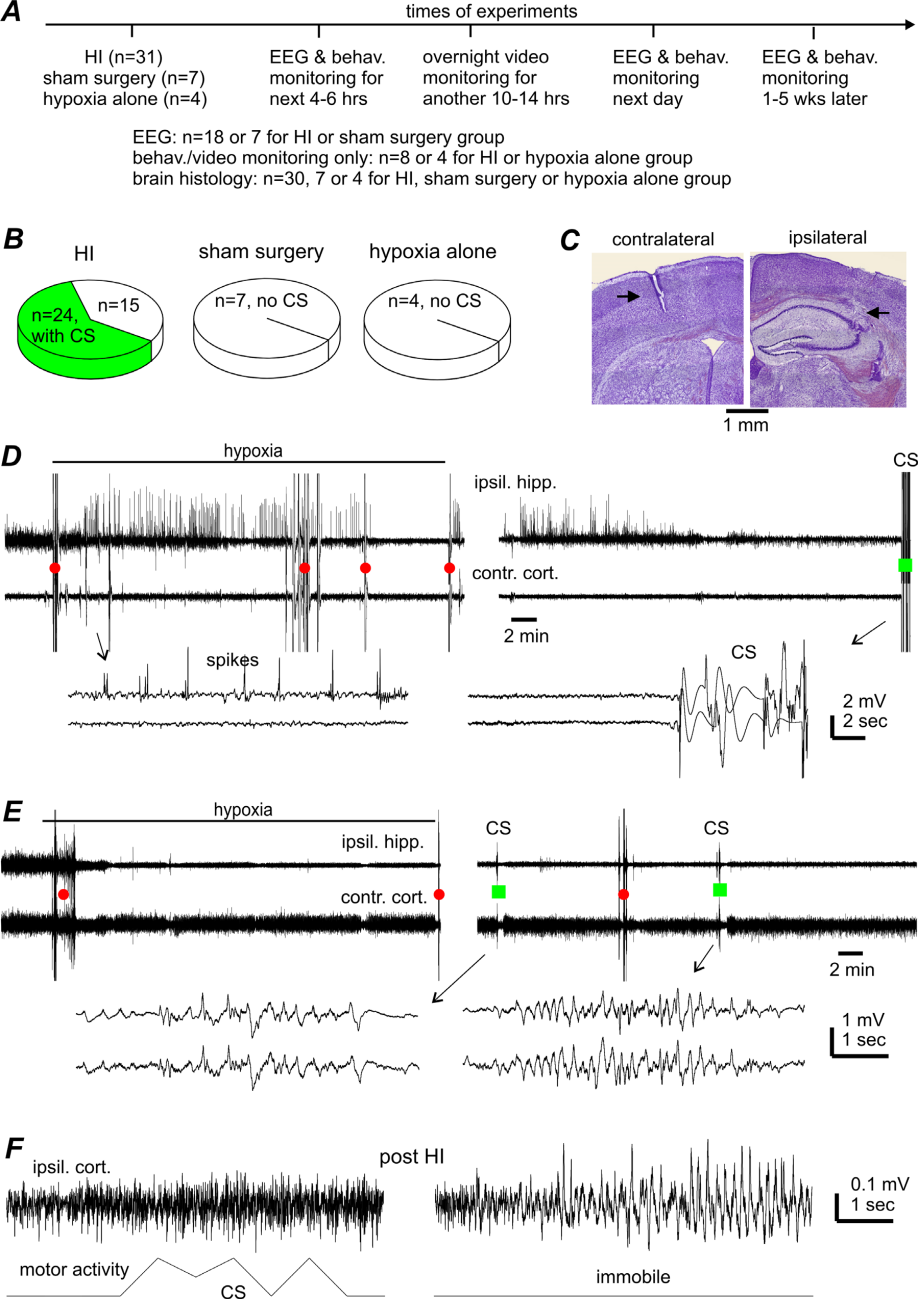
CS incidence and lack of CS-concurrent hippocampal-cortical EEG discharges. **A**, a schematic presentation of experimental manipulations and animal groups. **B**, CS incidences in aging mice following HI, sham surgery or hypoxia alone. **C**, representative brain histologic sections obtained from one aging mouse, showing the tracks (black arrows) of implanted EEG electrodes in the ipsilateral hippocampus (right) and contralateral cortex (left). Similar observations were made in other 9 animals to confirm the location of implanted EEG electrodes. **D-E**, representative EEG traces were collected from 2 aging mice before, during, and shortly after hypoxia. Tethered recordings were made from the ipsilateral hippocampus (ipsil. hipp.) and contralateral parietal cortex (contra. cort.). Original data were treated with band-pass filtered (0.5–500 Hz) for illustration purpose. Red circles or green squares denote movement artifacts in the absence or presence of CS. Arrowed segments are expanded below, showing hippocampal spikes (D, left) and movement artifact-contaminated signals during CS (D, right and E, right). **F**, signals of ipsilateral cortical EEG (top) and gross motor activity (bottom) collected telemetrically from another aging mouse. Left, evident motor activity signals without corresponding EEG discharges during a CS event. Right, EEG spikes in the absence of motor activity signals (during immobility).

Electrode implantation and recordings were conducted as previously described [38, 47, 60–62]. For tethered EEG recordings, recording electrodes were implanted bilaterally into the ipsilateral hippocampus (bregma -2.3 mm, lateral 2.0 mm and depth 2.0 mm) and contralateral parietal cortex (bregma -0.6 mm, lateral 1.5 mm and depth 1 mm) [63]. Here and in the following text, the terms “ipsilateral” and “contralateral” describe the hemispheres relative to the common carotid artery occlusion. A reference electrode was placed in the frontal lobe area (bregma +1.2 mm, lateral 2.0 mm, depth 0.5 mm). Locations of implanted electrode tracks were verified retrospectively on brain histology (**Fig 1C**).

EEG signals were recorded using a dual-channel AC microelectrode amplifier (model 1800, AM Systems, Carlsborg, WA, USA). Signals were collected in a frequency bandwidth of 0.1–1000 Hz, amplified 1000 times and then digitized at ≥5 KHz (Digidata 1300; Molecular Devices, Sunnyvale, CA, USA). The Pclamp software (version 9 or 10; Molecular Devices) was used for data acquisition, storage, and analysis. For telemetric recordings, we used a system (Data Scientific International, St. Paul, MN, USA) that simultaneously recorded electrographic signals, core body temperature, and gross motor activity ([38, 64]; see also **S1 File**). Telemetric EEG signals were recorded only from the ipsilateral parietal cortex as only one bio-potential channel was available. The transmission rate of the telemetric EEG signals was 200 Hz.

All EEG recordings were performed in free-moving animals. To quantify EEG changes over time, we used the root mean square (RMS) of the EEG signals as the RMS has previously been established to be a sensitive measure of ischemic EEG suppression in adult mice following HI [38, 65] and in rats following hemorrhagic seizures [66]. The RMS calculations were made from multiple 30-sec EEG segments collected while animals were immobile, when the signals were minimally contaminated by movement-related artifacts.

### Detection of early-onset seizures

Baseline monitoring was performed 1–2 weeks after electrode implantation and consisted of continuous visual monitoring by experimenters and EEG recordings up to 6 hours. To detect early-onset seizures following HI, individual animals underwent continuous behavioral monitoring and EEG recordings initially for 4–6 hours post-hypoxia and then overnight video monitoring for an additional 10–14 hours [38, 47]. Behavioral and EEG monitoring was resumed the next day and then at later previously determined serial time points [38] when animal survival permitted.

Vigorous CS, with behaviors including fast running, jumping, barrel rolling and/or falling (loss of righting reflex) with limb spasms, were noted at the time of onset and time-stamped on EEG and video recordings. Equivocal convulsive behaviors including head nodding, jerking, shaking, unilateral turning, and vocalization were not classified as CS. Ictal-like EEG discharges were defined as repetitive single spike and poly-spike waveforms with durations ≥5 seconds and amplitudes ≥2 times that of the background signals [47, 60, 67].

### Anticonvulsants

Lorazepam and fosphenytoin were obtained in clinically available injectable forms (Sandoz Canada Inc. and Erfa Canada Inc., Quebec, Canada). These drugs were diluted in saline and administered via intra-peritoneal injections at dosages of 1.5mg/kg for lorazepam and 30mg/kg for fosphenytoin (equivalent to phenytoin 20mg/kg).

### Brain histology

Brain histological assessments were conducted as previously described [38, 47, 61–62]. Animals were anesthetized with an intra-peritoneal injection of sodium pentobarbital (70 mg/kg) and transcardially perfused with 10% neutral buffered formalin solution. The brain was removed and further fixed in 10% formalin with 20% sucrose. Frozen coronal sections (30 or 20 μm thick) were obtained throughout the brain using a cryostat microtome. Cresyl-violet staining was used in the majority of animals to examine gross brain injury at different post-ischemic time points [57, 68] and to identify the tracks of implanted EEG electrodes. Fluoro-Jade C (Histo-Chem, Inc., Jefferson, Arkansas, USA) staining was carried out in accordance with the manufacturer’s instructions and protocols established in our previous studies [38, 60]. Images were obtained using a Leica (DMRN) upright microscope and analyzed using the Image J software (National Institute of Health, USA). Quantifications were made from brain sections at 8 coronal levels (bregma 1.9, 1.2, 0.5, -0.2, -1.1, -1.5, -2.4 and -3.2 mm respectively) [38, 47, 56, 61].

To quantify regions with weak or hypochromic cresyl-violet staining, the brightness and contrast of the Leica microscope captured images were adjusted to clearly demarcate boundaries where they existed. Only regions that were clearly stained lighter than adjacent areas and with recognizable boundaries were quantified. Ipsilateral areas with hypochromic staining were normalized as a % of the total ipsilateral hemispheric area at 8 coronal levels, and the averages from multiple coronal levels were presented for each animal (**Table 1**, column 7). For ipsilateral brain injuries that were difficult to quantify due to excess tissue loss and/or a lack of clear boundaries between injured and healthy tissue, only the coronal levels at which the hypochromic staining/infarctions were observed were indicated (**Table 1**, column 8). To quantify both ipsilateral edema and atrophy, the area of the total ipsilateral hemisphere was normalized as a % of the corresponding contralateral hemisphere at the same coronal levels. Quantifications made at multiple coronal levels were averaged for each animal to adequately demonstrate the extent and individual variability of the brain injury observed.

**Table 1:** Ipsilateral brain injuries observed histologically from individual aging mice.

Individual animals examined	HIPP	LAT CORT	STR	TH	MB/ BST	Regions with hypo-chromic staining (% of ipsilateral hemisphere)	Coronal levels at which ipsilateral injury was observed (mm from bregma)
Post-CS treated, 24–48 hours post HI							
#AG09C	+	+	+	+	+	77.0±2.4%	
#AG09D	+	+	+			35.3±6.9%	
#AU21B	+	+	+	+	+	66.5±4.7%	
#NO20C	+	+	+	+	+	37.8±5.0%	
#NO20B	+	+		+			-1.1 to -3.2
#NO20D	+	+	+				-0.2 to -3.2
#SE11A	+	+	+	+	+	64.9±3.6%	
#AG52	+	+	+			44.4±4.6%%	
#AU21C	+	+					-1.1 to -3.2
Untreated/post-CS treated, 4–5 weeks post HI							
#SE11B	+	+	+				1.2 to -2.4
#SE27B	+	+	+				1.2 to -2.4
#09B	+	+					-1.5 to -3.2
#AU15	+	+	+	+			1.2 to -2.4
Prophylactically treated, 24–48 hours post HI							
#ET2	+	+	+	+	+		1.2 to -3.2
#ET5	+	+					-1.5 to -3.2
#AG18	+	+					-1.5 to -2.4
Prophylactically treated, 4–5 weeks post HI							
#ET4	+	+	+				1.2 to -3.4
#AG15	+	+					-1.1 to -2.4
#AG19	+	+	+				0.5 to -2.4
#AG20			+				1.2 to 0.5
#AG22	+	+	+				1.2 to -2.4
#AG23	+	+	+				1.2 to -2.4

Ipsilateral brain injury was recognized through hypochromic staining when examined at 24–48 hours post-HI or cystic infarctions at 4–5 weeks post HI. Column 1: Animal ID of each aging mouse examined. Columns 2–6: injured brain structures indicated by ‘+’. Abbreviations: HIPP—hippocampus; LAT CORT—lateral cortex; STR—striatum; TH—thalamus; MB/BST—midbrain and brainstem areas. Column 7: ipsilateral regions with hypochromic staining where clearly recognized boundaries were present and quantifiable in 6 animals. These regions were measured at 8 coronal levels (bregma 1.9, 1.2, 0.5, -0.2, -1.1, -1.5, -2.4 and -3.2 mm respectively) and normalized as a % of total ipsilateral hemispheric area. The mean±SE from multiple coronal levels were presented for each animal. Column 8: ipsilateral brain injury observed for other animals in which injury margins were difficult to demarcate. For these animals, the coronal levels at which hypochromic staining or infarctions were observed were indicated for each animal instead.

Fluoro-Jade positive cells counts were made in the striatal, dorsal hippocampal, and caudolateral cortex at three coronal levels (bregma 0.5 mm, -1.5 mm, -3.2 mm). These areas were selected due to the greater extent of brain injury observed in these regions on cresyl-violet staining. Images were acquired under 20x magnification, resulting in an imaging field of 0.13 mm^2^. Fluoro-Jade positive cells in the entire field were counted, and cell counts from each of the three coronal levels were averaged for each animal [38].

### Statistical tests

SigmaStat software (Systat Software Inc., San Jose, California, USA) was used for statistical analysis. A Student’s t-test or Mann-Whitney rank sum test was used for two group comparisons. A Chi-square or Fischer exact test was used for rate comparisons. For multiple group comparisons, one-way ANOVA or one-way ANOVA on ranks was used, followed by a multiple comparison Dunn’s test versus baseline control or a Holm-Sidak multiple comparison pairwise test. Data were presented as the mean and standard error of the mean (SEM) throughout the text and figures. Statistical significance was set at p<0.05.

## Results

### General features of convulsive seizures (CS) observed from aging mice following HI

We utilized 39 aging mice in the experimental HI group, 7 in the sham surgery group and 4 in hypoxia alone group. **Fig 1A** outlines the three different experimental groups.

CS were observed in 24/39 aging mice following HI (**Fig 1B**), behaviorally identified as jumping, fast running, barrel-rolling, and/or falling (loss of the righting reflex) with limb spasms (**S1 Video**). Equivocal convulsive behavior including head nodding, jerking, shaking, unilateral turning, and abnormal vocalization were not classified as CS but were frequently seen in animals with CS. All CS occurred suddenly without an appreciable prodrome. However, animal handling or moderate auditory stimuli (such as cage knocking or nearby clapping) were sometimes able to trigger these CS. The CS observed were classified as acute, with an average latency (time from the termination of hypoxia to onset of the first CS) of 33.6±8.6 min (range: 5–120 min, n = 24). Seizure recurrence was common with an average interval of 21.8±5.5 min (range: 3–75 min) between the first and second CS. While all animals with CS received anticonvulsive treatment as per our animal care guidelines (see below), typically 1–4 CS recurred in animals during overnight video monitoring, approximately 6–15 hours post-HI. These observations suggest that aging mice may exhibit multiple CS or status epilepticus-like condition even hours after the anticonvulsive treatment (see Discussion).

A number of the remaining 15 aging mice without CS exhibited the equivocal convulsive behaviors described above. These were observed inconsistently within a 2-hour window immediately post-HI but not afterwards. No CS or any convulsive behaviors were seen in any control mice following either a sham surgery or hypoxia alone (n = 7 or 4, **Fig 1B**).

A total of 12 aging mice in the HI group underwent tethered EEG recordings from the ipsilateral hippocampus and contralateral cortex, with early-onset CS seen in 8 of these mice. No ictal-like discharges were observed in these animals up to 6 hours post-HI including during the CS (**Fig 1D and 1E**). However, EEG signals during the CS were contaminated with movement-related artifacts, which may have masked potential discharges (**Fig 1D**). To circumvent this limitation, we used telemetric EEG recordings, which are minimally affected by movement artifacts [38, 64] due its cable-free recording mechanism. Of the 6 aging mice that underwent post-HI telemetric EEG recordings from the ipsilateral cortex, 3 mice demonstrated post-HI CS, all in the absence of corresponding cortical discharges (**Fig 1F**, left). The lack of detectable EEG discharges during CS was unlikely to be due to technical limitations since our tethered EEG recordings were able to detect ipsilateral hippocampal spikes during and/or shortly after hypoxia (amplitude 1–4 mV, 5–30 spikes per min, from 4 seized animals; **Fig 1D**) and our telemetric recordings were similarly able to detect intermittent cortical spikes 10–18 hours post-HI (3–5 spikes per sec and durations of 2.4±0.2 sec; from the 3 animals with CS; **Fig 1F**, right). Instead, the lack of cortical discharges corresponding to these CS suggests the possibility that generation of these early-onset seizures may involve deeper subcortical structures.

### CS development closely associated with severe brain injury

**Fig 2:**
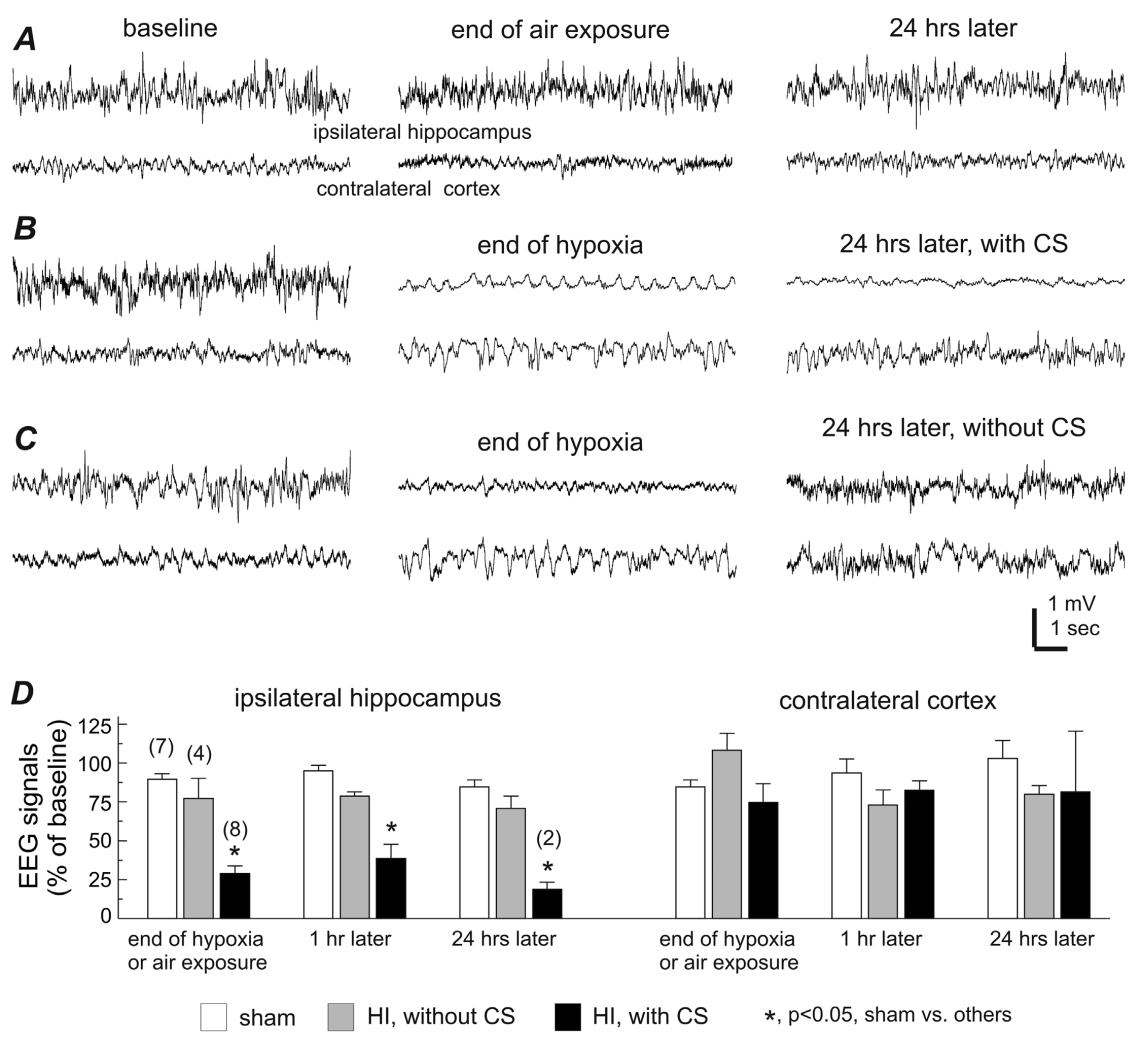
Decreases of ipsilateral EEG signals in aging mice with early-onset CS. **A**-**C**, representative EEG traces collected from 3 aging mice during baseline monitoring (left), at the end of ambient air exposure or hypoxia (middle), and 24 hours later (right). Tethered recordings were made from the ipsilateral hippocampus and contralateral parietal cortex for each animal. Note the ipsilateral EEG suppression in the animal with post-HI CS (B), recovered ipsilateral EEG in the animal without CS (C) and the lack of EEG suppression in the control animal (A). **D**, 30-sec EEG segments were collected during baseline monitoring, at the end of either hypoxia or ambient air exposure, then at 1 hour, and 24 hours following either a sham operation or HI. The root mean square (RMS) of the EEG signals was calculated and normalized as a percentage of the baseline RMS. Animals are grouped as sham controls and post-HI with and without CS. Data (mean±SE) for the ipsilateral hippocampus (left) and the contralateral cortex (right) are presented separately. *, p<0.05, sham control vs. others, one way ANOVA.

EEG signal suppression is believed to be a sensitive measure of brain ischemia [25–26, 69–70]. Previous work from our lab showed that early decreases in amplitude of the ipsilateral hippocampal EEG signals are closely associated with CS development in adult mice following HI [38] and aging mice following MCAO [47]. We observed a similar phenomenon here. In the 8 aging mice with CS that underwent tethered EEG monitoring, the EEG signals of the ipsilateral hippocampus, but not the contralateral cortex, were significantly suppressed at the end of hypoxia and 1 hour later (to 29% and 39% of the baseline level respectively when compared to the sham controls, p<0.05; **Fig 2A, 2B and 2D**). In comparison, in 4 aging mice without CS, there were no significant changes in the post-HI ipsilateral hippocampal EEG compared to the sham controls (**Fig 2C and 2D**). These observations, along with the short CS latency described above, suggest a close association between brain ischemia and subsequent CS development. However, there was no significant correlation between the ipsilateral EEG suppression and CS latency (r≥0.288, n = 8; p≥0.480, Pearson correlation), suggesting that a more complex, multifactorial process may be implicated in determining CS onset in individual mice.

**Fig 3:**
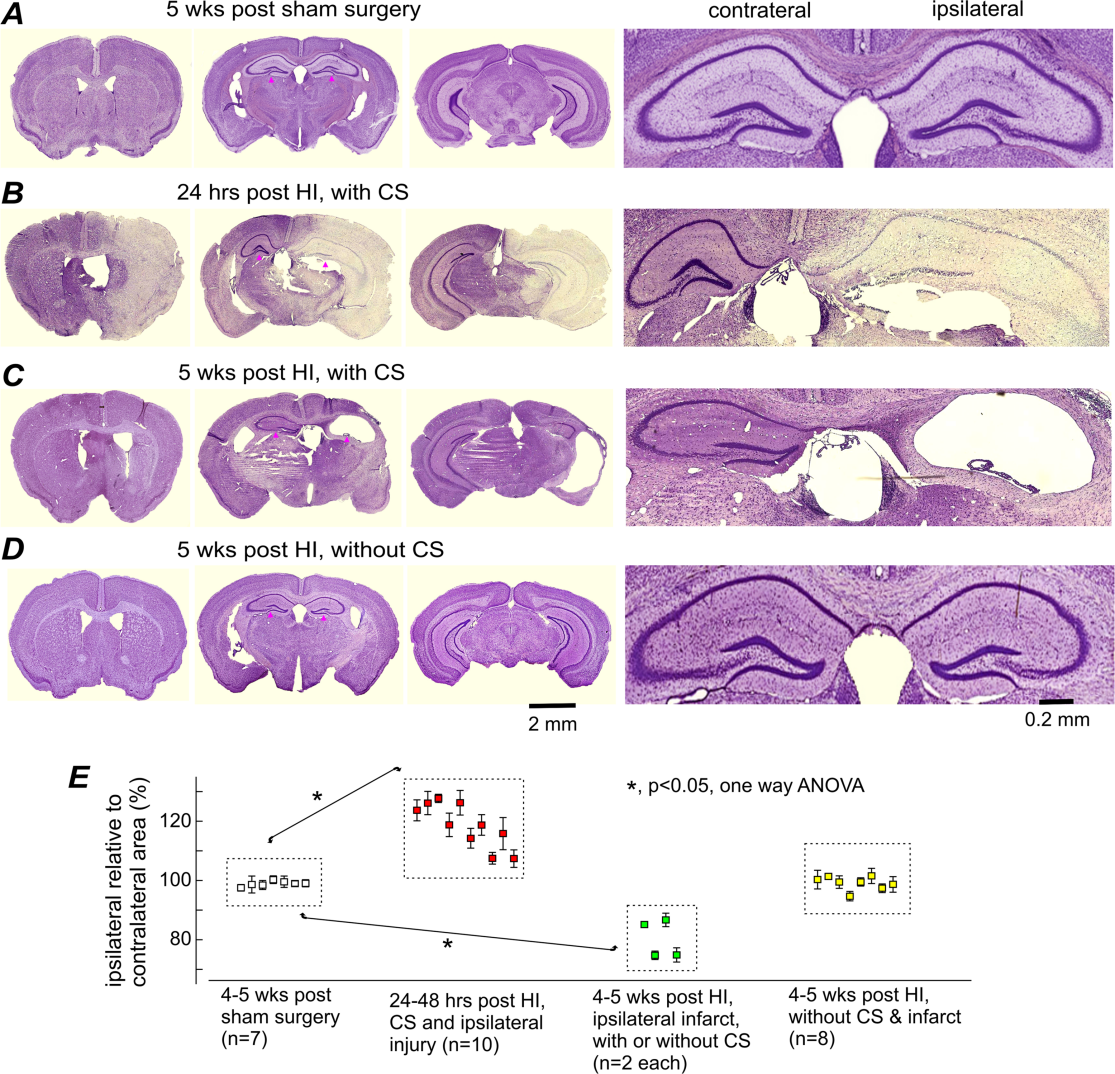
Ipsilateral brain injury or infarctions in aging mice with post-HI CS. **A**-**D**, images of cresyl violet-stained brain sections were obtained from 4 aging mice. Left, low power views at three coronal levels. Right, magnified views of the dorsal hippocampal areas (denoted by black dots on low power view). Time of histologic preparation, experimental group, and CS occurrence were indicated for each animal above their respective low power images. **E**, ratios of ipsilateral relative to contralateral hemispheric areas were obtained at 8 coronal levels and averaged for each animal. Data (%, mean±SE) for individual animals were pooled together according to the indicated experimental groups. *, p<0.05, sham controls vs. other experimental groups, one way ANOVA.

Cresyl violet-stained brain sections were obtained from aging mice euthanized either 24–48 hours post-HI, 4–5 weeks post-HI, or 4–5 weeks following control manipulation. Gross brain injury was analyzed at 8 coronal levels (bregma 1.9 mm to -3.2 mm) [38, 47, 56], and quantification from multiple coronal levels were averaged for each animal to describe the extent and individual variability of the brain injury. Euthanization 24–48 hours post-HI was mandatorily carried out in accordance with animal care guidelines when mice presented with refractory CS and/or in poor physical condition. At 24–48 hours post-HI, ipsilateral brain injury was denoted by regions of weak or hypochromic staining (a surrogate measure of cell injury), and by an enlarged ipsilateral to contralateral hemispheric area (indicative of ipsilateral edema; **Fig 3B**). These features were evident in all 10 aging mice with early-onset post-HI CS that underwent successful histological processing. Failure of histological processing in the remaining animals with CS was due to inadequate brain fixation as a result of spontaneous death, loss of friable tissues during frozen sectioning, or loss of tissue during staining. Quantifiable areas of hypochromic staining (with distinct, well-demarcated areas that could be contoured) were primarily in the striatum, hippocampus, lateral cortex, and midbrain/brainstem and occupied 35–77% of the total ipsilateral hemisphere in the 6 animals examined (**Fig 3B**; **Table 1**). The ratio of the total ipsilateral to contralateral hemispheric surface area was increased significantly to 109–132% that of the sham controls (**Fig 3E**) due to extensive ipsilateral edema at this early post-injury time point.

At 4–5 weeks post-HI, gross brain injury was recognized as structural deformities, cavities, and dark areas of scar tissue in the ipsilateral hemisphere, which we collectively refer to as cystic infarctions (**Fig 3C**). These infarctions were evident in 4/12 animals examined, both with and without early-onset CS (n = 2 each). The ratios of the ipsilateral to contralateral hemispheric areas were significantly decreased to 75–87% that of the sham controls (**Fig 3E**) due to widespread ipsilateral atrophy. The remaining 8 animals without CS did not show any cystic infarctions or a significant difference in ratios of the ipsilateral to contralateral hemispheric area when compared to the sham controls (**Fig 3A, 3D and 3E**). The lack of infarction and atrophy, along with the aforementioned lack of EEG suppression post-HI, suggests that these CS-free animals may have experienced a lesser degree of brain ischemia.

**Fig 4:**
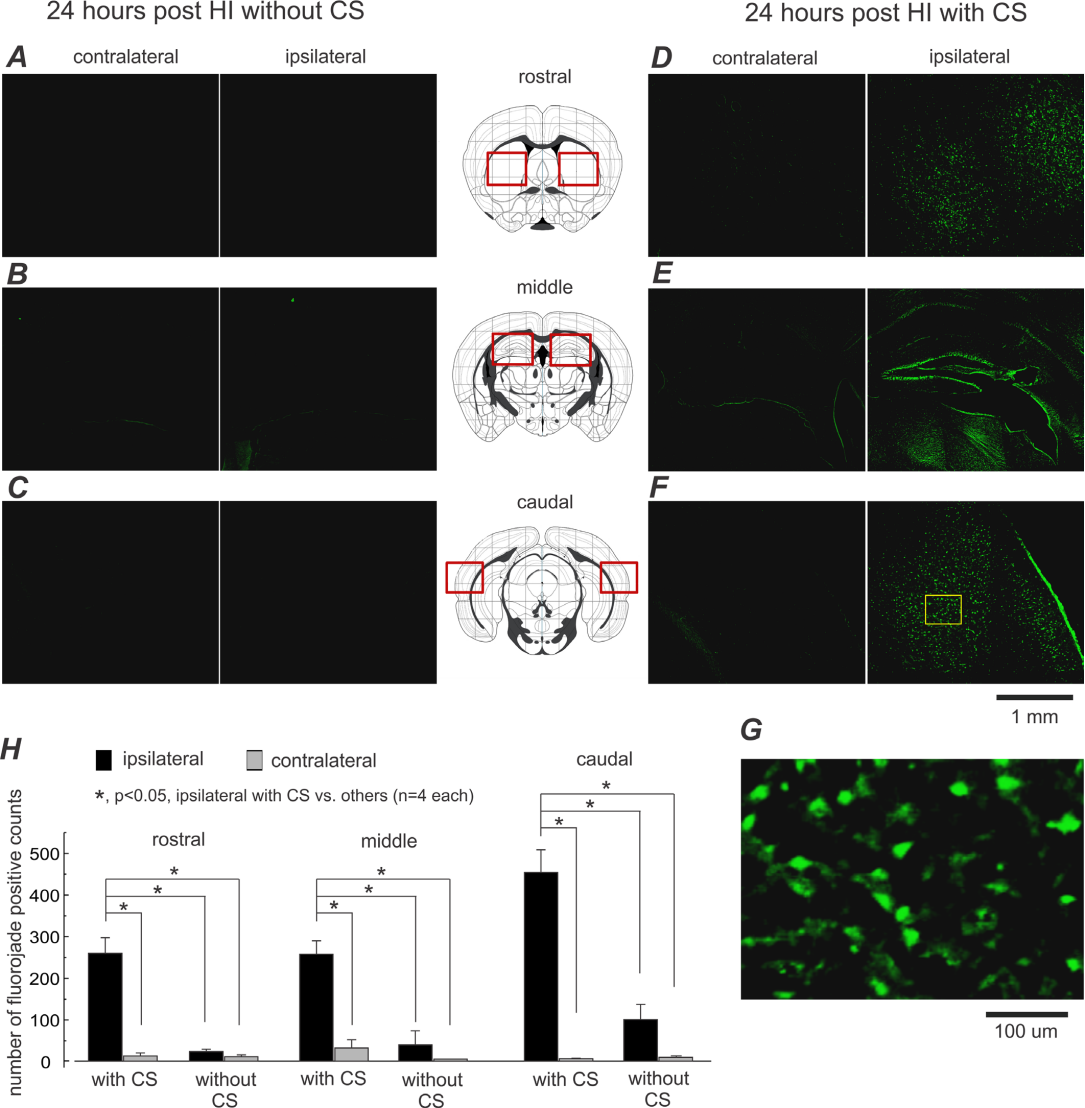
Abundant Fluoro-Jade positive cells in aging mice with post-HI CS. **A-F,** representative images were collected from 2 aging mice 24 hours following HI, one with CS (D-F, right), the other without (A-C, left). Brain regions in which Fluoro-Jade positive cells were analyzed are indicated diagrammatically in middle column (by red squares). **G**, an enlarged image was taken from a selected cortical area (indicated by a yellow square in F). **H**, regional counts of Fluoro-Jade positive cells in 8 post-HI aging mice with and without CS (mean±SE; n = 4 in each group). *, p<0.05, ipsilateral cell counts in animals with CS vs. contralateral cell counts and cell counts in animals without CS, one way ANOVA.

Fluoro-Jade positive signals, a marker of degenerating cells [71], were examined in 8 aging mice 24 hours post-HI. The number of ipsilateral Fluoro-Jade positive cells was significantly greater in animals with CS (**Fig 4D–4F and 4G**) than in CS-free animals (n = 4 each; **Fig 4A–4C and 4G**). The Fluoro-Jade positive cells were concentrated in the striatum, hippocampus, and lateral cortex (**Fig 4D–4F**), all in the ipsilateral hemisphere, consistent with the injury pattern seen on cresyl violet staining (**Fig 3B**).

In order to further elucidate the relationship between CS development and brain injury, we correlated CS occurrence with EEG and histologic outcomes. Severe brain injury was denoted as post-HI suppression of the ipsilateral EEG signal to ≤50% of the baseline level (**Fig 2B`and 2D**) and/or the presence of gross ipsilateral injury or infarctions on brain histology (**Fig 3B and 3C**). Evidence of severe brain injury was present in all 24 animals with acute CS but only in 2/10 CS-free animals (p<0.001, Odds ratio test). Therefore, early-onset CS appeared to develop exclusively in aging mice with severe brain injury, although not every animal with severe brain injury developed CS.

### Effects of anticonvulsive treatments

#### Post-CS treatment

Aging mice with acute CS were treated with clinically appropriate anticonvulsants as per our animal care guidelines. In the first set of treatment experiments, lorazepam and fosphenytoin (at 1.5 mg/kg and 30 mg/kg, intra-peritoneal injections) were given after the first two CS observed, while CS-free animals were untreated. We will subsequently refer to this treatment protocol as ‘post-CS treatment’ in the manuscript below. All 24 animals with early-onset CS described above received post-CS treatment. Lorazepam and fosphenytoin were injected at 52.2±5.2 min post-HI (range: 28–90 min, except one animal at 129 min post-HI). Post-CS treatment provided temporary seizure control for approximately 4–6 hours. However, CS invariably recurred later during overnight video monitoring (approximately 6–18 hours post HI, 1–4 CS events in each animal, n = 14).

**Fig 5:**
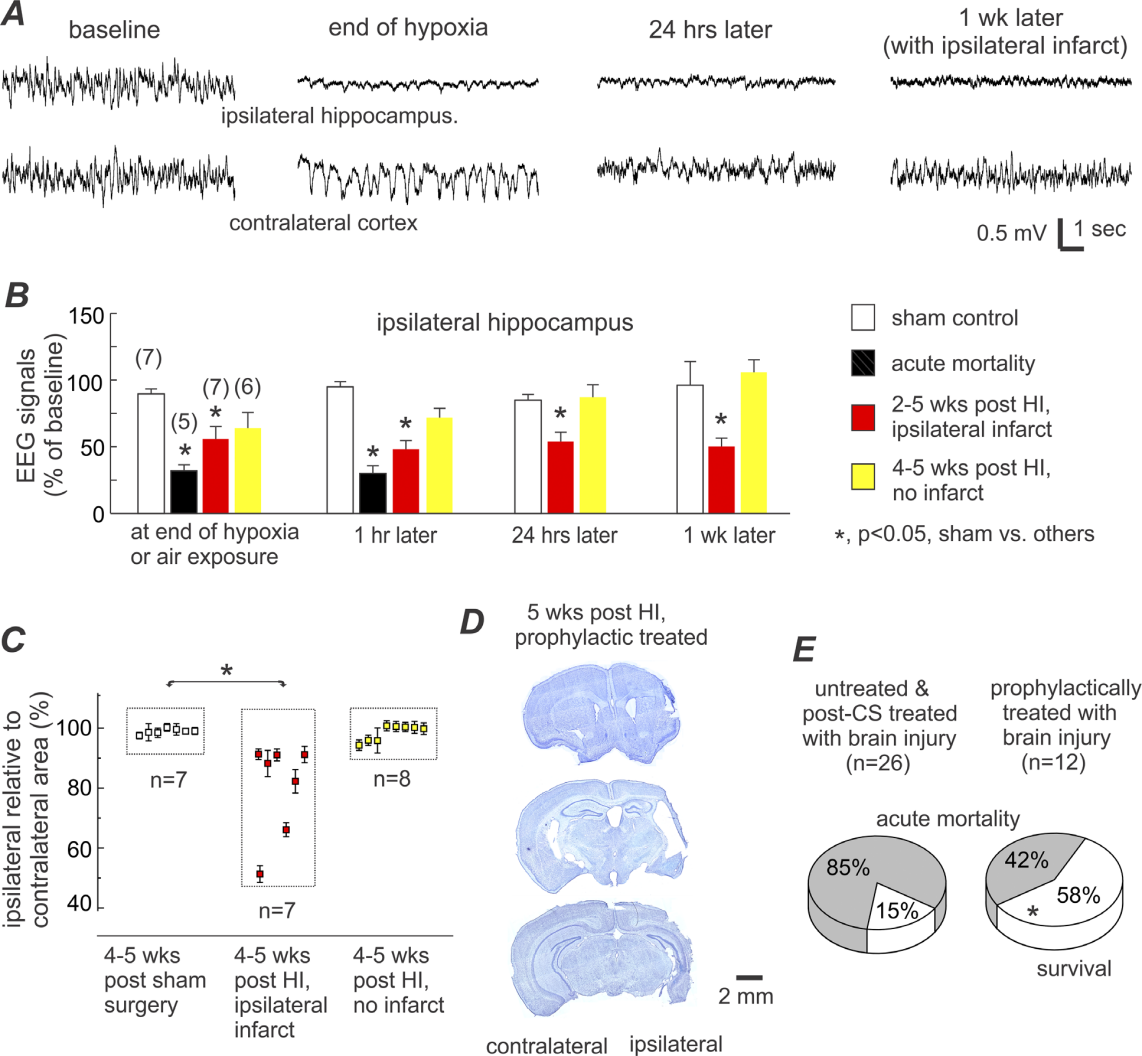
EEG and histological assessments and survival rate in prophylactic treated aging mice. **A,** representative EEG traces were collected from a prophylactically treated aging mouse. Tethered recordings made from the ipsilateral hippocampus (top trace) and contralateral parietal cortex (bottom trace). Traces from left to right: baseline signals, at the end of hypoxia, 24 hours post-HI, and 1-week post-HI. **B**, quantification of EEG signals in sham control and prophylactically treated aging mice. Only the changes in the ipsilateral hippocampal signals are presented for brevity. Numbers of animals examined in different time points are indicated in parentheses. *, p<0.05, sham controls vs. other experimental groups, one-way ANOVA. **C**, ratios of ipsilateral relative to contralateral hemisphere areas were measured at 8 coronal levels and averaged for each animal. Data (%, mean±SE) for individual animals were pooled together according to the indicated experimental groups. **D**, representative images of brain sections were obtained from another prophylactically treated aging mouse. Histologic assessments were conducted 5 weeks post-HI, showing ipsilateral infarctions and decreased ipsilateral hemispheric area at three coronal levels. **E**, survival rates in two groups of aging mice under the indicated experimental conditions. Acute mortality was defined as spontaneous death or mandatory euthanization within 48 hours post-HI, and survival referred to animals that lived 4–5 weeks post-HI in acceptable physical condition. *, p = 0.026, prophylactically treated vs. untreated/post-CS treated, Fisher exact test.

Survival was poor in the post-CS treated aging mice: 8 animals died during overnight video monitoring, 14 underwent mandatory euthanization the next day due to their poor physical condition, and only 2 survived to 5 weeks prior to euthanization for brain histology. The survival rate was therefore 2/24 (8%) for post-CS treated animals (**Fig 5E**). In comparison, of the 15 CS-free, untreated aging mice, 11 survived for 4–5 weeks including 2 with evident ipsilateral infarctions, and other 4 in good physical condition were euthanized 24 hours post-HI as controls for Fluoro-Jade staining. In the following text, the term “acute mortality” will be used to describe animals that either died spontaneously or underwent mandatory euthanization within 48 hours post-HI (see criteria for euthanization above).

#### Prophylactic treatment

Previous studies have shown that prophylactic anticonvulsive treatment can be effective in suppressing early-onset post-ischemic seizures, reducing both brain injury and acute mortality in adult rats and mice following either MCAO or HI [29, 33, 38]. Therefore, we decided to test this principle in aging mice. HI episodes were conducted as described above in an entirely separate cohort of aging mice (n = 21). The mean duration of hypoxia (27.2±0.9 min) did not significantly differ from that (27.8±1.1 min) of the main experimental cohort described above (p = 0.676). In the prophylactic treatment group, lorazepam and fosphenytoin (same doses as stated above) were injected within 5 min following termination of hypoxia, prior to the occurrence of any CS. Of the 21 prophylactically treated animals, 5 exhibited recurrent CS during overnight video monitoring and subsequently encountered acute mortality. Of the remaining 16 animals, 3 died 10–12 days post-HI while the remaining 13 survived for 4–5 weeks prior to euthanization for brain histological processing.

Brain injury in the prophylactically treated cohort was analyzed in an identical manner as described above in the post-CS treatment cohort. In the 5 animals that encountered acute mortality, the EEG signal of the ipsilateral hippocampus was decreased to 32% and 30% of the baseline level at the end of hypoxia and 1 hour later (**Fig 5A and 5B**) respectively, which were comparable to the decreases seen in aging mice from the post-CS treatment cohort (**Table 2**). Of the remaining 16 prophylactically treated animals surviving beyond the acute period, 7 demonstrated significant ipsilateral EEG suppression (**Fig 5B**), evident ipsilateral cystic infarcts, and decreased ratios of the ipsilateral to contralateral hemispheric area (**Fig 5C and 5D**). The ipsilateral to contralateral hemispheric area ratios in the prophylactic treatment cohort did not significantly differ from that seen in the untreated/post-CS treated animals described above (**Table 2**).

**Table 2:** EEG and histological measures from aging mice in the untreated/post-CS treated or prophylactically treated cohort.

**A**	Post-CS treated	Prophylactic treated
Ipsilateral hippocampal EEG changes in animals with CS (% of baseline)		
At end of hypoxia	28.9±4.9 (n = 8)	32.0±4.4 (n = 5)
1 hour later	38.7±9.0 (n = 8)	30.0±5.8 (n = 5)
24 hours later	18.8±4.6 (n = 2)	23.9 (n = 1)
**B**	Untreated	Prophylactically treated
Ipsilateral EEG changes in animals without CS and infarctions (% of baseline)		
At end of hypoxia	77.2±13.0 (n = 6)	63.9±11.8 (n = 6)
1 hour later	78.8±2.6 (n = 6)	71.7±7.0 (n = 6)
24 hours later	70.8±7.9 (n = 4)	87.0±9.4 (n = 6)
**C**	Post-CS treated	Prophylactic treated
Ipsilateral/contralateral area ratios (%) for individual animals with ipsilateral infarctions		
	85.2±0.1	51.3±2.8
	74.8±1.4	91.3±1.8
	86.7±2.3	88.2±4.4
	74.9±2.4	91.1±2.0
		66.1±2.3
		82.3±3.9
		91.2±2.7

Post-CS and prophylactic anticonvulsive treatments were conducted in two separate cohorts of aging mice (n = 24 and 21 respectively). **Panel A**: Changes in ipsilateral hippocampal EEG signals were analyzed in 8 and 5 animals with CS from these two cohorts respectively. Ipsilateral hippocampal EEG signals were normalized as a % of the baseline. The numbers of animals examined at different post-HI time points are indicated in the parentheses. **Panel B**: ipsilateral EEG signals were analyzed in untreated animals and prophylactically treated animals (n = 6 each) that did not exhibit CS and ipsilateral infarctions on later histological assessment. Data are similarly presented in Panel A. **Panel C**: The ratio of the ipsilateral to contralateral hemispheric areas measured in histological brain sections. These measurements were made in 4 untreated/post-CS treated animals and 7 prophylactically treated animals 4–5 weeks post-HI. There were no significant group differences in any EEG or histological measures (p>0.05, t test or Mann-Whitney Rank Sum Test).

Based on the above EEG and histological assessments of severe brain injury, only 12 prophylactically treated animals may be deemed as having undergone sufficient brain ischemia (5 animals with ipsilateral EEG suppression and subsequent acute mortality and 7 animals that survived to 4–5 weeks post-HI with ipsilateral infarcts on histology). Therefore the corrected survival rate, taking only these animals into account, is 7/12 or 58% for the prophylactic treatment group, which was significantly greater than the survival rate for the post-CS treated animals (2/24 or 8%; p = 0.006) and for all animals with evident ipsilateral brain injury with or without post-CS treatment (4/26 or 15%; p = 0.026; **Fig 5E**). This suggests that prophylactic treatment may effectively inhibit CS development and improve survival in aging mice with severe brain injury.

## Discussion

Three main findings emerged from this study: 1) HI can induce early-onset CS in aging mice; 2) CS development is closely associated with severe brain injury and acute mortality; 3) Prophylactic anticonvulsive treatment can inhibit CS development and improve survival in post-HI aging mice.

### Limitations and complications of the HI model

As with other models of brain ischemia, the HI model has its limitations. One major concern is that systemic hypoxia may alter cardiorespiratory activity in addition to producing the intended brain ischemia, thereby complicating interpretation of the model’s outcomes [72]. Therefore, to evaluate the potential systemic effects of hypoxia on aging mice, we measured core body temperature, electrocardiographic signals, and regional cerebral blood flow. In response to the hypoxia, aging mice showed only minor changes in body temperature and heart rate but substantial decreases of regional cerebral blood flow in the ipsilateral hemisphere (**S1 File**). These observations are consistent with previous findings in adult animals [38, 56] and further suggest that the induced brain ischemia is the likely primary cause of HI brain injury in aging mice.

Another complication with the HI model is the variability of the brain injury. In our present experiments, evidence of ipsilateral brain injury was observed in 67% aging mice (excluding the prophylactic treatment cohort) following HI, but not in the remaining 33% of animals. The presence of brain injury in some animals but not in others may be partly due to the relatively moderate degree of hypoxia (8% O_2_ for 30 min) used in this protocol. Hypoxic episodes of longer duration or at a lower percentage of O_2_ can increase the rate and severity of HI-induced brain injury [54–56], but they may not be well tolerated by aging mice. Another contributing factor to the variability may be the hypoplastic posterior communicating arteries often observed in C57 black mice as these, when present, have been associated with more extensive brain injury in models of focal and global brain ischemia [73–77]. This anatomical defect may have contributed to the severe brain injury and the higher prevalence of early-onset CS in our model than what has been documented clinically [3–10, 27].

Our recent work revealed the presence of early-onset non-convulsive seizures (NCS) in aging and adult C57 black mice following MCAO [47]. These NCS occurred prior to the CS and manifested robust hippocampal-cortical EEG discharges in the absence of evident convulsive behavior. Unlike in the MCAO model, acute NCS or ictal-like EEG discharges were not observed from adult [38] or aging mice following HI. This may be largely due to the significant ipsilateral EEG suppression observed during and shortly following the hypoxic episode. However, other potential EEG discharges at later post-HI time points, such as those previously demonstrated in neonatal and immature rats [78–79], remain to be examined in our HI model. Future experiments that will utilize routine telemetric EEG and video monitoring [80–81] will be critical for studying these phenomena.

### Early-onset CS are a common symptom of severe brain ischemia in C57 black mice

In our recent work that modeled early-onset seizures in aging mice following MCAO, we observed a strong association between CS development and brain injury [47]. A similar phenomenon was noted in these experiments as post-HI CS appeared to develop exclusively in aging mice with EEG and/or histologic evidence of severe brain injury. In addition, CS observed following either HI or MCAO presented almost identically from a behavioral standpoint and remained consistent with the generalized tonic-clonic convulsions previously described in other rodent models [82]. Other similarities include the fact that CS in both the HI and MCAO models could be triggered by animal handling or moderate auditory stimuli but neither model showed CS-concurrent hippocampal-cortical EEG discharges. Together these data suggest that early-onset CS are a common feature of severe brain ischemia in aging mice.

The specific mechanisms underlying the pathogenesis of early-onset, post-HI CS remain to be investigated. Subcortical structures have long been implicated in seizure generation and propagation in animal models [83–84]. CS with rapid running, jumping, and/or barrel-rolling have been observed in rodent models of brainstem electrical kindling [85–86] and audiogenic seizures during ethanol withdrawal [87]. In these models, the CS were thought to involve or affect brainstem structures based on local EEG discharges or spike activity. Areas within the brainstem have also been implicated in seizure genesis in a rat model of systemic bicuculline injections (a GABAA receptor antagonist) [88]. The post-ischemic CS we describe share similarities with those induced by brainstem kindling or auditory stimuli during ethanol withdrawal [85–87] in their behavioral features including the tactile/auditory triggering of these seizures. In addition, post-HI CS were not associated with any concurrent hippocampal/cortical EEG discharges but rather only with ipsilateral EEG suppression (**Fig 1**). Furthermore, in aging mice that survived for 4–5 weeks post-HI, we failed to identify any gross brain injury in the midbrain-brainstem areas despite evident injury in the cortical, hippocampal, and striatal regions (**Table 1**). In light of these findings, we hypothesize that compromised descending inhibition due to brain ischemia may facilitate the generation of CS from deeper subcortical structures, particularly brainstem loci. Future experiments that include EEG recording from specific brainstem areas and detailed histological assessment of subtle cellular brainstem injury may help test this hypothesis.

### Survival-improving effects of prophylactic anticonvulsive treatment

Lorazepam and fosphenytoin are recommended treatments for status ‎epilepticus clinically [89–90]. We combined ‎lorazepam and fosphenytoin in order to synergistically enhance GABAergic inhibition and reduce Na^+^ channel mediated hyperexcitability. These two drugs were applied either after two observed CS (post-CS treatment) or within 5 min of hypoxia termination, prior to CS occurrence (prophylactic treatment). Overall, post-CS treatment offered transient seizure control but failed to improve animal survival as 22/24 treated animals still encountered acute mortality. The poor outcomes might be partly attributable to the development of status epilepticus-like conditions [91–92] which tend to be generally resistant to standard anticonvulsive therapy [93] as 1–4 CS were observed from post-CS treated animals during overnight video monitoring. The exacerbation of both the underlying brain injury and the already poor physical condition of these animals by the CS may play a contributing role as well. In comparison, 7/12 prophylactically treated animals with EEG and/or histologic evidence of ipsilateral brain injury survived for 4–5 weeks post-HI, a significant improvement from the survival of the post-CS treated group. These observations corroborate the results of previous studies in adult animals [29, 33, 38] and further suggest that early anticonvulsive treatment may improve survival in ischemic aging mice.

However, it must be acknowledged that the variability of the induced brain ischemia may have confounded the results of prophylactic treatment in aging mice. At the end of hypoxia and 1 hour later, the ipsilateral EEG signals seemed to show decreases of smaller magnitude in the 7 surviving animals with infarctions seen on later histology compared to the 5 animals that encountered acute mortality (**Fig 5B**). Although these differences were not statistically significant, we cannot exclude the possibility that the 7 surviving animals may have simply experienced a less severe ischemic insult, thereby predisposing them to better outcomes irrespective of treatment effect. In addition, the sample size of animals that survived up to 4–5 weeks post-HI with ipsilateral infarctions were small in both the post-CS and prophylactic treatment groups. Therefore, caution must be taken when comparing survival outcomes between the two groups (**Fig 5E**) as well as comparing related EEG and histologic measures (**Table 2**). Furthermore, the half-life of fosphenytoin or lorazepam in human plasma is approximately 12 hours [89–90]. It is therefore possible that decreased plasma levels of fosphenytoin and lorazepam in post-CS treated animals might have contributed to CS recurrence during overnight video monitoring. The effects of repeated or continuously applied anticonvulsive treatments, similar to the strategy used clinically for controlling status epilepticus [89–90] remain to be evaluated in these animals. Taking these caveats into consideration, our present observations still suggest that prophylactic anticonvulsive treatments may improve survival in aging mice with severe brain ischemia.

The clinical relevance of prophylactic anticonvulsive treatment is debatable as currently there is insufficient evidence to support or reject this in patients with cardiac arrest or ischemic stroke [8–10; 15, 18, 20–21, 94]. Concerns center around the adverse effects of anticonvulsive drugs and age related alterations in anticonvulsant pharmacokinetics, which may complicate stroke outcomes and post-stroke recovery. The limited availability of aging mice did not permit the testing of different anticonvulsants at varied doses in our present experiments. Nevertheless, although optimal dosing could not be established, it is unlikely that treatment with lorazepam and fosphenytoin played a role in exacerbating acute mortality as prophylactic treatment was shown to improve survival outcomes. Interestingly, treatment with low doses of levetiracetam has been shown to improve hippocampal memory function in elderly patients with mild cognitive impairment [95–96] and in aged animals experimentally [97–99]. It remains to be seen whether prophylactic or early treatment with levetiracetam [37, 100] or other newer antiepileptics [101] can be similarly beneficial for ischemic aging animals.

### Influences of ages on CS genesis and anticonvulsive treatment outcomes

Previous work from our lab [38] and the present experiments conducted similar HI episodes in adult and aging C57 black mice (4–9 and 18–20 months-old respectively). Early-onset CS observed from these two groups of mice appeared to be comparable in regards to convulsive behaviors, latency, incidence, the lack of concurrent cortical/hippocampal EEG discharges and the early decreases (at the end of hypoxia and 1 hour later) of ipsilateral EEG signals. These similarities suggest that adult and aging mice may share common mechanisms that underlie CS development in the early phase of HI process. However, existing data are inadequate to reveal potential age differences in brain injury particularly at later post-HI times largely because of poor survival of ischemic aging mice.

In the previous study [38], adult mice were treated with diazepam+phenytoin. In our present experiments, aging mice were treated with lorazepam+fosphenytoin as lorazepam has a longer duration of effect compared to diazepam and fosphenytoin has a better side effects profile than phenytoin [89–90]. Despite the use of different anticonvulsants, prophylactic treatment in both the adult and aging cohorts proved to be more effective than post-CS treatment at inhibiting CS and improving survival. However, compared to adult mice, a higher percentage of aging mice encountered acute mortality, presented in poor physical condition (such as with prolonged immobility and/or compromised dietary intake) or died within 2 weeks of prophylactic treatment, reflecting their relative intolerance to brain injury and more rapid associated physiological decline post-insult [57–58]. Further strategies that can improve animal survival, particularly for aging animals, are needed before better characterization of ischemic brain injury, late-onset epileptic seizures, and other comorbidities in ischemic aging mice can be completed.

## Supporting Information

S1 File Measurements of body temperature, heart rate and regional cerebral blood flow from post-HI aging mice. (PDF)

S1 Video Post-HI CS observed from an aging mouse. (MPG)
