# Supplementary material for: Early-Onset Convulsive Seizures Induced by Brain Hypoxia-Ischemia in Aging Mice: Effects of Anticonvulsive Treatments
Source: PLoS One. 2015 Dec 2;10(12):e0144113. doi: 10.1371/journal.pone.0144113 (PMC4668036; doi:10.1371/journal.pone.0144113)
Supplement: S1 File — (PDF) [file pone.0144113.s001.pdf]

S1 File: Body temperature, heart rate and regional cerebral blood flow measured from post-HI aging mice.

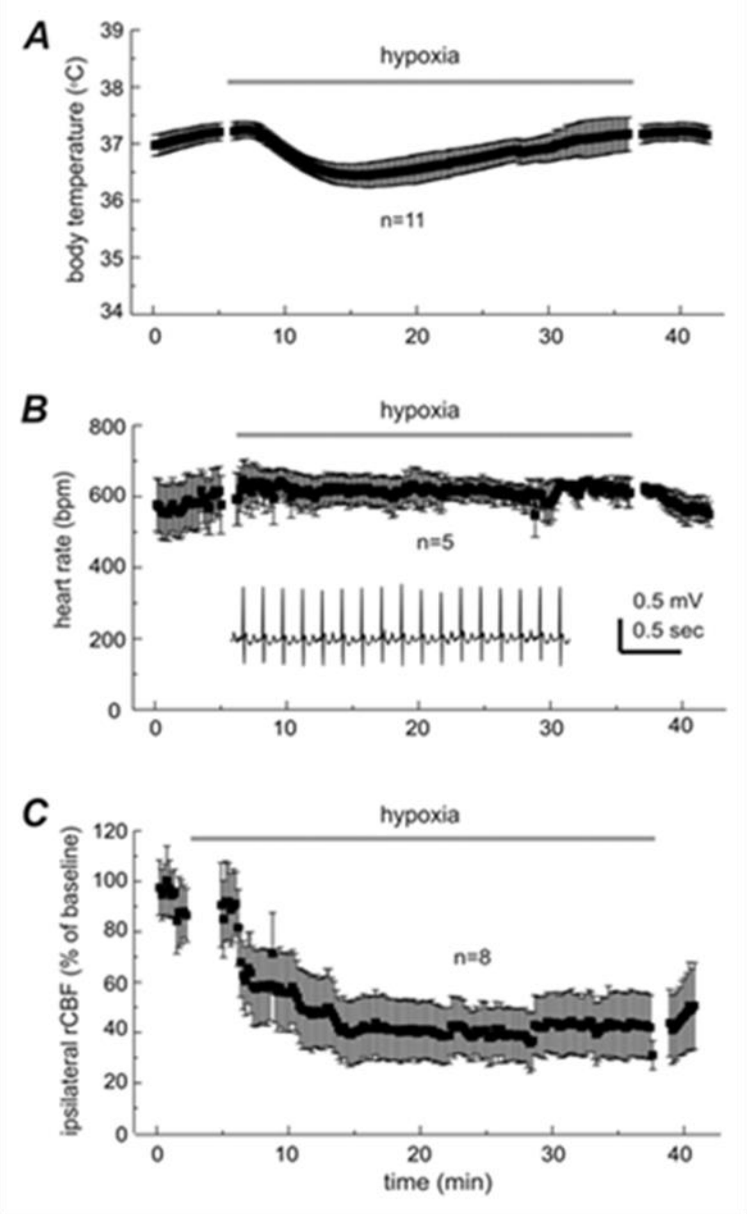

Figure legend

Data were collected from aging mice after occlusion of the right common carotid artery. The duration of hypoxia was indicated above each plot by a horizontal line. Measurements

(mean±SE) from individual animals were grouped and plotted vs. time. A, intra-peritoneal temperature was measured continuously and averaged every 10 seconds. B, heart rates were calculated from ECG signals and averaged every 30 seconds. Insert, a representative ECG segment collected from one aging mouse at the end of the hypoxia. C, regional cerebral blood flow (rCBF) was measured from the ipsilateral hemisphere, averaged every 30 seconds and then normalized as % of the baseline in individual animals.

Data details and Methods

Intra-peritoneal temperature and ECG signals were recorded telemetrically in free-moving aging mice as previously described [1]. Measured temperatures were 36.6±0.24°C during baseline monitoring and 37.1±0.01°C at 1.5 hours after occlusion of the common carotid artery and before hypoxia (n=11). There was a small but significant drop from the baseline temperatures by approximately 0.5°C during the hypoxia (S1 FigA). Lower body temperatures were also observed 1 hour later (35.4±0.33°C; $p<0.05$, post hypoxia vs. baseline, one way ANOVA). However, all the temperature measures following the hypoxia were within physiological temperature range (34-38°C) as per previous telemetric studies in adult mice [1-4]. Post-HI CS were observed in 6/11 animals examined.

Regular ECG signals were observed during baseline monitoring and following hypoxia (n=5 aging mice; S1 FigB). Calculated heart rate was 527.1±22.9/min during baseline monitoring and 578.4±3.4/min after occlusion of the common carotid artery and before hypoxia. Heart rate was significantly increased from baseline to 622.9±2.2/min at the end of hypoxia and 559.7±3.9/minute at 1 hour later ($p<0.05$, one way ANOVA). These increases might reflect a compensatory reaction of aging mice to increase cardiac output following hypoxic stress. Post-HI CS were observed in 3/5 animals examined.

A laser Doppler system (PF5010, Perimed, Järfälla, Sweden) was used to measure regional cerebral blood flow (rCBF) through the skull1. Ipsilateral rCBF signals were recorded from free-moving aging mice using a protocol modified from a previous study [5]. Briefly, the skull surface was surgically exposed under 2% isoflurane anesthesia, and a mouse-specific probe (MTB 500) was glued onto it in an area from bregma -2.2 to -2.8 mm and lateral 1.5 to 2.2 mm. Baseline rCBF signals were collected before occlusion of the common carotid artery. The rCBF signals at 1.5-hours after the common carotid artery occlusion was 93.4±6.5% of the baseline level. A modified hypoxic episode with 15% O2 in the first 5 min and 8% O2 in next 30 min was used. This modification was to reduce animal movement and thereby also rCBF signal fluctuations in the early phase of the hypoxic challenge. The rCBF signals collected during 8% O2 hypoxia were analyzed as animals were largely immobile during this period. Ipsilateral rCBF signals declined substantially during the hypoxia (n=8 aging mice; S1 FigC). Measured at the end of hypoxia and at 15 min and 1 hour later, ipsilateral rCBF signals were 46.7±9.1%, 52.2±13.5%, and 48.0±6.3% of the baseline level, significantly lower than the level measured after the common carotid artery occlusion and prior to hypoxia ($p<0.05$, one way ANOVA). Post-HI CS were observed in 6/8 animals examined.

References for supporting information

1) El-Hayek YH, Wu CP, Chen R, Al-Sharif AR, Huang S, Patel N, et al. Acute post-ischemic seizures are associated with increased mortality and brain damage in adult Mice. Cerebral Cortex 2011; 21: 2863-2875.

2) Weiergräber M, Henry M, Hescheler J, Smyth N, Schneider T. Electrocorticographic and deep intracerebral EEG recording in mice using a telemetry system. Brain Res Protoc 2005;14: 154-164.

3) Arraj M, Lemmer B. Circadian Rhythms in heart rate, motility, and body temperature of wild-type C57 and eNOS knock-out mice under light-dark, free-run, and after time zone transition. Chronobiol Int 2006; 23:795-812.

4) Ovechkin AV, Tyagi N, Sen U, Lominadze D, Steed MM, Moshal KS, et al. 3-Deazaadenosine mitigates arterial remodeling and hypertension in hyperhomocysteinemic mice. Am J Physiol Lung Cell Mol Physiol 2006; 291: L905-L911.

5) Masuki S, Nose H. Increased cerebral activity suppresses baroreflex control of heart rate in freely moving mice. J Physiol 2009; 587: 5783-5794.
